# Diagnostic accuracy of an artificial intelligence-based software in detecting supernumerary and congenitally missing teeth in panoramic radiographs

**DOI:** 10.1093/ejo/cjaf054

**Published:** 2025-07-05

**Authors:** Miltiadis A Makrygiannakis, Kostis Giannakopoulos, Argyro Kavadella, Dimitrios Paraskevis, Eleftherios G Kaklamanos

**Affiliations:** School of Dentistry, European University Cyprus, 6 Diogenous str., Nicosia 2404, Cyprus; School of Dentistry, National and Kapodistrian University of Athens, 2 Thivon str., Athens 11527, Greece; School of Dentistry, European University Cyprus, 6 Diogenous str., Nicosia 2404, Cyprus; School of Dentistry, European University Cyprus, 6 Diogenous str., Nicosia 2404, Cyprus; School of Dentistry, European University Cyprus, 6 Diogenous str., Nicosia 2404, Cyprus; Medical School, National and Kapodistrian University of Athens, 75 Mikras Asias str., Athens 11527, Greece; School of Dentistry, European University Cyprus, 6 Diogenous str., Nicosia 2404, Cyprus; School of Dentistry, Aristotle University of Thessaloniki, Aristotle University of Thessaloniki Campus, Thessaloniki 54124, Greece; Hamdan Bin Mohammed College of Dental Medicine, Mohammed Bin Rashid University of Medicine and Health Sciences, Dubai Healthcare City, Dubai P.O. Box: 505055, United Arab Emirates

**Keywords:** supernumerary teeth, hypodontia, artificial intelligence, diagnocat™

## Abstract

**Background/Objectives:**

Recent advances in AI have enabled its application in dentistry. This study assessed the diagnostic accuracy of an AI-based model (Diagnocat™) in detecting congenitally missing and supernumerary teeth on panoramic radiographs.

**Materials/Methods:**

Three groups of 50 orthopantomograms each—control, congenitally missing, and supernumerary teeth—were evaluated by two human observers and Diagnocat™. Diagnostic performance was compared using the Wilcoxon Signed Rank and McNemar’s tests. Agreement was measured using Cohen’s Kappa, and diagnostic metrics (sensitivity, specificity, positive predictive value (PPV), and negative predictive value (NPV)) were computed using IBM SPSS 29.0.

**Results:**

For congenitally missing teeth, Cohen’s Kappa indicated strong agreement (0.91); however, significant differences were found in the diagnostic performance (p < 0.01). The model exhibited 84.7% sensitivity, 100.0% specificity, 100.0% PPV, and 99.4% NPV. For supernumerary teeth, the agreement was moderate (Kappa = 0.60), with significant differences in the diagnostic performance (p < 0.001). Sensitivity was 43.9%, while specificity, PPV, and NPV were 100.0%, 100.0%, and 98.9%, respectively.

**Limitations:**

Using convenience sampling and a retrospective design may affect generalizability and applicability.

**Conclusions/Implications:**

Although the AI-based model shows promise, it is not yet able to replace human assessment as the standard for detecting missing and supernumerary teeth in panoramic radiographs.

## Introduction

A panoramic radiograph is one of the most commonly used imaging techniques in dentistry. It provides a two-dimensional image of the maxilla, mandible, teeth, and surrounding anatomical structures [1–3]. It is characterized by several advantages, including a relatively low radiation dose compared to full mouth series of periapical radiographs or CBCT, a quick imaging process, and minimal inconvenience to the patient [4]. Since accurate diagnosis based on the interpretation of these radiographs requires specialized knowledge and expertise [5], automated assessment of panoramic radiographs could assist clinicians in their routine daily practice.

Artificial Intelligence (AI) represents one of the latest breakthroughs in technology that could prove valuable in this area. In recent years, the dentistry field has seen significant advancements in the development and implementation of AI models. Integrating AI into dentistry aims to help professionals further improve oral healthcare services. A recently published white paper highlights the potential of these models to assist with various functions, including image analysis, radiograph interpretation, diagnosis using neural networks, data synthesis, material information, and clinical techniques aimed at optimizing outcomes. Furthermore, AI applications encompass the management of patient records, forensic dentistry, orthodontics, periodontology, endodontics, caries diagnosis, treatment planning, and even enhancing communication and interaction with patients [6].

A variety of tools and software have been developed by several companies to meet a wide range of diagnostic imaging needs. These systems are tailored for different types of two-dimensional imaging, including intraoral, panoramic, and cephalometric images, as well as CBCT scans. They offer a broad array of diagnostic applications, from segmenting teeth to producing comprehensive reports of radiographic findings [7]. In this context, several studies have explored the effectiveness of AI models, particularly convolutional neural networks (CNNs), in identifying teeth and pathological findings in dental radiographs. So far, efforts have been directed toward detecting dental lesions [8, 9], caries [10, 11], periodontal bone loss and periodontitis [12], as well as root fractures [13].

Diagnocat™ (Diagnocat Inc, San Francisco, CA, USA) is one of these models, based on an AI algorithm and marketed as an ‘All-in-one artificial intelligence software for 2D and 3D’ [14]. According to the company, Diagnocat’s™ market positioning is characterized by its innovative use of AI to enhance dental diagnostics, its cloud-based platform that facilitates seamless integration into dental practices, and its emphasis on improving patient communication and outcomes [14]. Since its inauguration in 2018, over the past five years, research has been conducted using real data from around the globe for its development, leading to its continuous refinement [15, 16]. It has been investigated for dental diagnostics, both for two-dimensional X-rays and CBCT [15, 16]. Relevant exploration of the diagnostic capabilities of Diagnocat™ on CBCTs demonstrates that, among other functions, it can identify teeth, quantify the number of roots and canals, assess crown condition [16], perform tooth segmentation [17], and evaluate endodontic treatment outcomes [18]. Additionally, research has shown that Diagnocat™ can aid in caries diagnosis following the assessment of intraoral radiographs [19].

Research related to Diagnocat™’s diagnostic efficiency on panoramic X-rays has been limited. The overall reliability of the model to identify dental conditions on panoramic radiographs was evaluated by Zadrożny and colleagues [20] and Orhan et al. [21]. Additionally, two further studies aimed to assess the diagnostic accuracy of Diagnocat™ in detecting periapical lesions in panoramic X-rays and CBCTs, as well as in evaluating endodontic treatment outcomes in dental pantomograms [22, 23]. To date, there is no research assessing the diagnostic accuracy of Diagnocat™ in detecting anomalies related to the number of teeth.

Given the current absence of evidence regarding the effectiveness of Diagnocat™ in analysing panoramic X-rays, this study sought to assess its accuracy in identifying congenitally missing and supernumerary teeth by comparing its performance with evaluations provided by expert clinicians. The null hypothesis stated that there is no difference in the detection ability of congenitally missing or supernumerary permanent teeth between the AI model and the expert assessment.

## Materials and methods

### Study sample

The present study received approval from the Institutional Committee on Bioethics and Ethics at European University Cyprus (EUC ETHICS COMMITTEE 2025-23). All stages of the methodology were carried out in accordance with relevant guidelines for diagnostic accuracy studies [24].

Due to the lack of prior knowledge about the efficacy of this model in detecting anomalies in the number of permanent teeth—whether congenitally missing or supernumerary—a power calculation was not feasible. A retrospective analysis was conducted using a convenience sample of radiographs obtained for routine dental or orthodontic assessments. Patients aged 8–34 years, that represent the majority of the orthodontic population [25], without a history of craniofacial malformations, permanent tooth extractions, or previous orthodontic treatment, were included. Ultimately, three sets of radiographs were evaluated, each consisting of 50 anonymized orthopantomograms: one set with no anomalies in the number of permanent teeth (control group) and two sets featuring either congenitally missing [range: 1–8] or supernumerary [range: 1–2] permanent teeth (excluding third molars).

### Assessment of radiographs and data collection

Two experienced orthodontists, one Associate Professor in Orthodontics (EGK) and one PhD candidate (MAM), served as a reference standard and independently assessed the included radiographs for the presence of supernumerary teeth or the congenital absence of permanent teeth under standardized viewing conditions, recording their findings separately. All images were mixed and blinded, and the evaluators were not aware of the presence of anomalies beforehand. Although it had initially been planned that consensus would be sought in cases of disagreement, and if unable to reach an agreement, the respective radiograph would be removed from the sample, no such instances occurred, and inter-examiner reliability was 100%. The same sets of radiographs were uploaded to the Diagnocat™ server for analysis. The software automatically processed the images and generated a comprehensive report of detected teeth.

A standardized Excel form was used for each radiograph to record findings from both assessment methods. The form included fields for expert assessments and AI model diagnoses, as well as variables for evaluating true positives and false positives, true negatives, and false negatives for the AI model compared to the experts’ assessments.

### Statistical analysis

The statistical analysis focused on comparing the tooth anomaly detection potential of Diagnocat™ with the assessments made by expert clinicians. Cohen’s Kappa was utilized to evaluate the agreement level between Diagnocat™ and the expert clinicians. The Kappa statistic was computed on a per-tooth basis, where each tooth was assessed individually for the presence or absence of anomalies (congenitally missing or supernumerary teeth) by both the experts and the AI model. Additionally, McNemar’s tests were conducted to identify differences in evaluations between the two methods. The differences in the mean number of missing or supernumerary teeth per radiograph were analysed using appropriate statistical tests based on the data distribution. If the data followed a normal distribution, the parametric t-test for paired samples was applied to assess the differences. For data that did not meet the assumption of normality, the non-parametric Wilcoxon Signed Rank test was utilized. The normality of the data was evaluated using the Shapiro-Wilk test. Additionally, sensitivity, specificity, positive predictive value, and negative predictive value were computed. All statistical analyses were conducted using IBM SPSS v.29.0. The significance level for all hypothesis testing procedures was set at α = 0.05 (p ≤ 0.05).

## Results

The potential of the AI model to detect congenitally missing or supernumerary permanent teeth was evaluated by comparing its performance against the expert’s assessment. Table 1 presents the distribution of congenitally missing teeth alongside the teeth that were positively or negatively diagnosed by the AI model. For the detection of congenitally missing teeth, Cohen’s Kappa coefficient was 0.91, indicating a high level of agreement between the two methods. However, McNemar’s test showed a significant difference in their assessments (p < 0.01). The mean number of missing teeth per radiograph identified by the AI model and the experts were compared, with the results shown in Table 2. The difference between the means of teeth identified per radiograph was analysed using the Wilcoxon Signed Rank test, revealing a statistically significant difference (p < 0.01). The sensitivity and specificity of the Diagnocat™ compared to expert clinicians’ assessment were estimated to be 84.7% and 100.0%, respectively. Additionally, the positive predictive value (PPV) and negative predictive value (NPV) were found to be 100.0% and 99.4%, respectively (Table 3).

**Table 1.** Distribution of congenitally missing teeth.

	Maxillary							Mandibular							Total
	Central incisor	Lateral incisor	Canine	First premolar	Second premolar	First molar	Second molar	Central incisor	Lateral incisor	Canine	First premolar	Second premolar	First molar	Second molar	
**AI Positive**	4	24	0	1	5	0	3	1	3	0	3	30	1	8	**83**
**AI Negative**	0	5	2	1	4	0	2	0	0	0	0	1	0	0	**15**
**Total**	4	29	2	2	9	0	5	1	3	0	3	31	1	8	**98**

**Table 2.** Comparison of the average number of missing teeth (per radiograph).

Method of detection	Observations	Missing teeth per radiograph (Mean, SD, SE)		
Expert assessment	50	1.96	1.58	0.223
Diagnocat™	50	1.66	1.24	0.175

SD: Standard deviation; SE: Standard error.

**Table 3.** Comparison of positive and negative diagnoses for identifying congenitally missing teeth.

	Diagnocat™		
Expert assessment	Positive	Negative	Subtotal
**Positive**	83	15	98
**Negative**	0	2702	2702
**Subtotal**	83	2717	2800

Tables 4–6 present the distribution of supernumerary teeth and their classification based on location, morphology, and eruption status [26], alongside the teeth that the AI model positively or negatively identified. For supernumerary teeth, Cohen’s Kappa coefficient was 0.60, indicating moderate agreement. The Wilcoxon Signed Rank and McNemar’s tests also revealed significant differences (p < 0.001) (Tables 7 and 8). The sensitivity, specificity, PPV, and NPV of the AI method in comparison to the expert assessment were 43.9%, 100.0%, 100.0%, and 98.9%, respectively (Table 8).

**Table 4.** Distribution of supernumerary teeth.

	Maxillary				Mandibular				Total
	Incisors	Canines	Premolars	Molars	Incisors	Canines	Premolars	Molars	
**AI Positive**	17	0	1	0	3	0	2	2	**25**
**AI Negative**	19	0	2	1	1	0	8	1	**32**
**Total**	36	0	3	1	4	0	10	3	**57**

**Table 5.** Classification of supernumerary teeth, based on location.

	Location					Total
	Mesiodentes	Other incisors	Parapremolars	Paramolars	Distomolars	
**AI Positive**	6	14	3	0	2	**25**
**AI Negative**	5	14	9	2	2	**32**
**Total**	11	28	12	2	4	**57**

**Table 6.** Classification of supernumerary teeth, based on morphology and eruption status.

	Morphology			Eruption status		Total
	Conical	Tuberculate	Supplementary	Erupted	Non-erupted	
**AI Positive**	5	5	15	14	11	**25**
**AI Negative**	7	5	20	5	27	**32**
**Total**	12	10	35	19	38	**57**

**Table 7.** Comparison of the average number of supernumerary teeth (per radiograph).

Method of detection	Observations	Supernumerary teeth per radiograph (Mean, SD, SE)		
Expert assessment	50	1.14	0.35	0.05
Diagnocat™	50	0.50	0.54	0.08

SD: Standard deviation; SE: Standard error.

**Table 8.** Comparison of positive and negative diagnoses for identifying supernumerary teeth.

	Diagnocat™		
Expert assessment	Positive	Negative	Subtotal
**Positive**	25	32	57
**Negative**	0	2800	2800
**Subtotal**	25	2832	2857

Using the McNemar test for paired binary values, we calculated post-hoc power from the observed discordant pairs for both congenitally missing and supernumerary teeth detection. In the post-hoc power analysis for congenitally missing teeth (n = 15 discordant pairs; Table 3), the calculated power (alpha level = 0.05) was greater than 99.9%, indicating that the study was sufficiently powered to identify the observed difference (p < 0.001). Likewise, for supernumerary teeth (n = 32 discordant pairs; Table 8), the post-hoc power analysis showed a calculated power (alpha level = 0.05) exceeding 99.9%, which suggests that the study had adequate power to detect the observed difference (p < 0.001).

## Discussion

In daily clinical practice, diagnosing tooth anomalies related to number, such as supernumerary and congenitally missing teeth, relies on a combination of clinical examination and detailed inspection of X-rays. Detecting missing and supernumerary teeth is crucial, as it serves as an important parameter in treatment planning from both orthodontic, oral surgery, and prosthodontic perspectives. The prevalence of supernumerary teeth ranges between 1.2% and 6.0% for permanent teeth and between 0.3% and 0.8% for primary dentition [27–29]. Single supernumerary teeth are typically located in the maxillary anterior area, while multiple supernumerary teeth are most often observed in the premolar region [27, 30]. The congenital absence of permanent teeth, excluding third molars, shows a prevalence of 3%–10%, or approximately one in every 10–12 individuals, with a 3:2 female-to-male ratio [31–33]. Congenital absence occurs most frequently in the most distal tooth of a type, namely third molars, second premolars, and lateral incisors [34, 35].

Artificial Intelligence (AI) is a promising new technology that may be useful in dental diagnostics, particularly for tooth number anomalies. Deep learning algorithms, like Convolutional Neural Networks (CNNs), have demonstrated notable potential. Currently, there is very limited available data regarding the detection of tooth anomalies. In fact, only a small number of studies have focused on supernumerary teeth, impacted canines, and impacted teeth in general [17, 36–38]. However, validation studies, especially by independent research teams, are insufficient. One of the novel CNN architecture models is Diagnocat™ (Diagnocat Co. Ltd., San Francisco, CA). Thus far, relevant research conducted on this model has been used for purposes such as CBCT segmentation, 3D cephalometry, and caries detection [16, 17, 39]. The authors of this study did not find sufficient prior evidence regarding tooth number anomalies detection by this specific model.

This study found a high level of agreement between Diagnocat™ and expert assessments regarding the detection of missing teeth, but moderate agreement in identifying supernumerary teeth. Despite the strong agreement for AI-based methods compared to the expert assessments, a significant difference was noted in the mean number of missing teeth identified by both methods. The McNemar’s test also revealed a significant difference in their assessments, indicating that the two methods did not match perfectly. In the assessment of missing teeth, Cohen’s kappa indicated high overall agreement, while the significant McNemar’s test suggested substantial directional disagreement in the proportions of teeth correctly identified as missing compared to those erroneously identified as present. Although the two assessments generally agree, the AI model significantly underestimated true positive results, resulting in a sensitivity of 84.7%. The differences between Diagnocat™ and human assessments were more pronounced for supernumerary teeth, where in several cases the AI method failed to detect their presence. Although the marketing company does not disclose information about the algorithm behind their AI product, the varying levels of agreement in identifying missing and supernumerary teeth may suggest differences in design, training datasets, and performance characteristics for the two clinical scenarios.

The lack of clarity regarding the algorithmic structure and training datasets for the Diagnocat™ software is a widespread challenge seen in many commercial AI models used in dentistry. Since the company does not reveal particular technical specifications—such as the model type, training parameters, or the composition of the datasets—the software operates as a ‘black box’. This hampers clinicians’ and researchers’ capabilities to understand its diagnostic outcomes or to confirm the reliability of its results across various clinical situations. The lack of explainability could erode clinician confidence in AI applications, especially when their outputs differ from expected clinical results [40, 41]. Additionally, the failure to reproduce outcomes under similar conditions creates obstacles for maintaining scientific rigour and diminishes the model’s applicability in clinical settings. This concern is especially relevant in dentistry, where interpreting radiographs necessitates significant contextual understanding, and the model’s performance may be very susceptible to anatomical differences and image quality [42].

AI models frequently show lower accuracy when evaluated beyond their training environments, a situation termed domain shift [43, 44]. Additionally, lacking details about the demographics and clinical characteristics of the training dataset raises concerns regarding possible algorithmic biases. Studies indicate that AI models trained on limited or unbalanced datasets can consistently underperform for underrepresented groups or uncommon conditions [45, 46], such as supernumerary teeth. These challenges highlight the need for transparent AI development and validation processes to ensure equitable and trustworthy integration into clinical settings.

While the algorithm behind Diagnocat™ remains proprietary and its error-reporting mechanisms lack transparency, our analysis of AI misclassifications in the dataset revealed recurring patterns. In many instances, errors were associated with cases of mixed dentition, where the presence of both primary and permanent teeth may confuse the AI regarding tooth numbering or classification. Previous AI studies have documented similar diagnostic challenges in mixed dentition cases [35]. In our test sample, 59% of cases belonged to individuals in the mixed dentition category. Moreover, mistakes were more common in non-erupted teeth that may have suboptimal radiographic visibility. Teeth with ambiguous morphological features, such as those exhibiting atypical root or crown morphology, may also have contributed to these misclassifications.

Another relevant consideration is the potential challenge of detecting overlapping structures, which often occurs with supernumerary teeth. While no studies have been found that discuss the complications of detecting overlapping teeth in panoramic X-rays, evidence from other fields supports this idea. In the medical field, Hamanaka and Oda [47] examined AI’s potential to identify lung tumours in X-rays. However, when shadows overlapped with anatomical structures, both physicians and AI showed reduced diagnostic accuracy. To address this challenge, various approaches have been proposed, such as overlap-aware box selection that utilizes a predicted overlap map to determine which highly overlapping bounding boxes correspond to actual overlapping objects and should be retained rather than pruned [48]. In the field of forensics, a refined version of the YOLO (You Only Look Once) model has been utilized, integrating edge detection and image segmentation techniques to enhance the identification of overlapping shoeprints. By focusing on crucial boundary details between shoeprint textures and the surface, this method improved sensitivity and precision, achieving confidence levels above 85% for minimally overlapped images and maintaining over 70% for highly overlapped cases. Furthermore, heatmaps of convolution layers were produced to visualize how the network optimizes detection through these enhancements [49].

The AI model showed strong agreement and high specificity for both congenitally missing and supernumerary teeth; however, its sensitivity for detecting supernumerary teeth was notably low at 43.9%. This diminished sensitivity indicates that while Diagnocat™ is unlikely to generate false positives, it may overlook a significant number of existing supernumerary teeth. Missing these supernumerary teeth can result in complications such as impaction or root resorption in adjacent teeth, which can ultimately complicate orthodontic or surgical procedures [27, 50]. As a result, relying solely on Diagnocat™ for initial screenings could pose a risk of underdiagnosis, especially in cases involving supernumerary teeth. Nonetheless, its high specificity and negative predictive value imply that when the AI model identifies a supernumerary tooth, it is highly trustworthy. Thus, Diagnocat™ can act as a useful supplementary tool rather than a primary diagnostic method, particularly advantageous in routine reviews of panoramic radiographs to assist less experienced clinicians or in high-volume environments where a second opinion could be beneficial. The AI model required about half a minute to perform the analysis. However, in situations where there is clinical suspicion (such as delayed eruption or asymmetry), AI findings should be corroborated through expert radiographic interpretation to ensure a complete diagnosis.

In clinical practice, Diagnocat™ could be relevant as a supportive tool for diagnostic workflows, especially in orthodontics and paediatric dentistry, where anomalies in tooth number can greatly affect treatment decisions. A recent study evaluated orthodontists’ skills in identifying and addressing incidental findings on panoramic radiographs. The most common findings included impacted teeth, hypodontia, dense bone islands, and supernumerary teeth. Using the risk assessment tool, 35% of the findings were classified as highly significant. The overall agreement between orthodontists and oral and maxillofacial radiologists was fair, with a kappa value of 0.32 (95% CI: 0.30–0.34) [51]. It is worth mentioning that no AI models were utilized in this research.

Based on the aforementioned points, it is clear that even specialists face challenges in detecting incidental findings in radiographs, let alone dentists who have not advanced their clinical expertise. In daily orthodontic practice, incidental findings are relatively common, with prevalence rates ranging from 8.7%–96.3% [52–57]. Since orthodontists may be the first dental practitioners to prescribe and examine a panoramic X-ray, it is crucial to have all necessary resources for a comprehensive radiographic evaluation. Therefore, AI models such as Diagnocat™ could assist in this regard. However, regarding hypodontia and the presence of supernumerary teeth, further development is still required.

The diagnostic performance of non-commercial AI models in identifying congenitally absent and supernumerary teeth varied based on the condition analysed and the architecture employed (Table 9). For mesiodens detection, multiple models—like Inception-ResNet-v2 [58, 59], U-Net/ResNet50 [60], and YOLOv3 [61]—showed high diagnostic performance, with sensitivities ranging from 87.9% to 97.1%, and accuracies reaching up to 97.1%. Importantly, Ha et al. noted only a slight drop in external validation (accuracy: 89.8%, sensitivity: 87.9%), indicating the models’ generalisability. In contrast, models for absent teeth revealed greater variability. Kim et al. (2024b) reported moderate sensitivity (63.5%) and accuracy (73.8%) [62], whereas Chen et al. (2022) demonstrated better metrics (sensitivity up to 99%, accuracy up to 97.2%) using transfer learning with GoogLeNet [63]. For supernumerary teeth beyond mesiodens, performance tended to be more modest: Mine et al. (2021) found sensitivities around 85% and accuracy near 80%, suggesting that these anomalies present a diagnostic challenge for present AI systems [36].

**Table 9.** Diagnostic performance of non-commercial AI models in identifying congenitally missing and supernumerary teeth.

Study	Detection focus	Training/test dataset	AI architecture	Accuracy/sensitivity/specificity
Ahn et al., 2021 [59]	Mesiodens	1000/100	SqueezeNet ResNet-18 ResNet-101 Inception-ResNet-V2	Acc: 65.9%, Sens: 88.0% Acc: 82.0%, Sens: 76.0% Acc: 86.0%, Sens: 88.0% Acc: 88.0%, Sens: 90.0%
Chen et al., 2022 [63]	Missing	245/105	AlexNet GoogLeNet SqueezeNet	Acc: 92.9%; Sens: 99.0% Acc: 97.2%; Sens: 96.0% Acc: 92.9%; Sens: 98.10%
Ha et al., 2021 [61]	Mesiodens	612/Int: 130 & Ext: 118	YOLOv3	Internal: Acc: 96.2%, Sens: 95.4%, Spec: 96.9% External: Acc: 89.8%, Sens: 87.9%, Spec: 91.7%
Kim et al., 2022 [58]	Mesiodens	790/198	Inception-ResNet-v2	Acc: 97.1%, Sens: 97.1%
Kim et al., 2024a [60]	Mesiodens	750/100	U-Net/ResNet50	Acc: 92%, Sens: 95.0%
Kim et al., 2024b [62]	Missing	645/161	Inception-ResNet-v2	Acc: 73.8%, Sens: 63.5%, Spec: 81.4%
Mine et al., 2021 [36]	Supernumerary	176/44	AlexNet VGG16-TL InceptionV3-TL	Acc: 80.5%, Sens: 82.5%, Spec: 78.0% Acc: 802.3%, Sens: 85.0%, Spec: 79.8% Acc: 80.9%, Sens: 83.3%, Spec: 78.0%

Acc: accuracy; Ext: External; Int: Internal; Sens: sensitivity; Spec: specificity.

A significant limitation of this study is that Diagnocat™’s performance was assessed using a convenience dataset sourced internally. While this method is pragmatic and allows for thorough expert validation and controlled comparisons, it restricts the generalizability of our results. The radiographs included in this sample might not reflect the complete spectrum of clinical variability found in larger patient populations. The varying distribution of mixed dentition cases could have led to uneven diagnostic challenges for the AI model. Furthermore, differences related to gender in dental development and the prevalence of anomalies may have influenced the outcomes. Additionally, the radiographic equipment and imaging protocols—such as the manufacturer, image resolution, exposure settings, and patient positioning—may not fully represent the variability seen in other clinical environments. Collectively, these factors indicate that while the results provide valuable insights into Diagnocat™’s diagnostic capabilities, one should exercise caution when applying these findings to other clinical contexts. The retrospective nature of the study and its small sample size may also hinder broader applicability. Evaluating the model on additional datasets annotated for tooth number anomalies—preferably from various populations and imaging systems—could enhance understanding of its robustness and real-world applicability.

Furthermore, the proprietary nature of the Diagnocat™ model limited a comprehensive examination of its underlying algorithms, thereby restricting insight into the differences between AI-generated and human clinical evaluations. In practical terms, this underlines the necessity for expert clinical validation of Diagnocat™-generated diagnoses prior to their implementation in patient care. This limitation underscores a broader issue with AI integration in healthcare. We recommend that AI vendors adopt open science policies, including transparency about training datasets’ size, diversity, and origins, algorithm architectures, and external validation performance. Such transparency is essential for independent evaluations, regulatory assessments, clinician trust, bias detection, and ethical patient care. Standardized reporting frameworks, similar to CONSORT-AI extensions, could help improve the accountability and reliability of clinically relevant AI systems for researchers and users.

Additionally, the overlapping anatomical structures in panoramic radiographs may have impacted the accuracy of identifying supernumerary teeth, highlighting the need for further refinement of AI models to address these specific diagnostic challenges. AI models could benefit from improved training on developmental variants and ambiguous anatomy to more accurately represent the range of real-world cases.

Future developments in AI dental diagnostics should focus on developing explainable AI (XAI) systems that furnish visual or textual justifications for their predictions. Such systems may enhance clinicians’ understanding of AI’s decision-making processes, especially in borderline or ambiguous cases, thus increasing trust and promoting a safer implementation into clinical practices. Additionally, training AI models with varied radiographic datasets that include a broad range of anatomical variations—like mixed dentition, partially erupted teeth, impacted teeth, or morphologically atypical teeth—could improve diagnostic reliability in complex cases. Taking these steps is vital to ensure that AI tools evolve beyond simple accuracy measures to become clinically interpretable, generalizable, and ethically applicable in practical scenarios.

## Conclusions

In conclusion, our study revealed that the Diagnocat™ AI model showed high agreement with expert clinicians in detecting missing teeth, although it did not provide a perfect match regarding the number of missing teeth. The sensitivity was measured at 84.7%, indicating that further improvement in the AI model is still needed. When detecting supernumerary teeth, the AI model demonstrated moderate agreement with expert assessments, suggesting it may be less effective in identifying these anomalies. Overall, the AI model shows promise as a reliable tool, particularly in detecting congenitally missing teeth, although enhancements are required for the accurate detection of supernumerary teeth.

## Data Availability

The data are available from the corresponding author upon reasonable request as per relevant regulations.
